# Impact of Burnout on Self-Reported Patient Care Among Emergency Physicians

**DOI:** 10.5811/westjem.2015.9.27945

**Published:** 2015-12-11

**Authors:** Dave W. Lu, Scott Dresden, Colin McCloskey, Jeremy Branzetti, Michael A. Gisondi

**Affiliations:** * Northwestern University Feinberg School of Medicine, Department of Emergency Medicine, Chicago, Illinois; † University of Washington School of Medicine, Department of Emergency Medicine, Seattle, Washington

## Abstract

**Introduction:**

Burnout is a syndrome of depersonalization, emotional exhaustion and sense of low personal accomplishment. Emergency physicians (EPs) experience the highest levels of burnout among all physicians. Burnout is associated with greater rates of self-reported suboptimal care among surgeons and internists. The association between burnout and suboptimal care among EPs is unknown. The objective of the study was to evaluate burnout rates among attending and resident EPs and examine their relationship with self-reported patient care practices.

**Methods:**

In this cross-sectional study burnout was measured at two university-based emergency medicine residency programs with the Maslach Burnout Inventory. We also measured depression, quality of life (QOL) and career satisfaction using validated questionnaires. Six items assessed suboptimal care and the frequency with which they were performed.

**Results:**

We included 77 out of 155 (49.7%) responses. The EP burnout rate was 57.1%, with no difference between attending and resident physicians. Residents were more likely to screen positive for depression (47.8% vs 18.5%, p=0.012) and report lower QOL scores (6.7 vs 7.4 out of 10, p=0.036) than attendings. Attendings and residents reported similar rates of career satisfaction (85.2% vs 87.0%, p=0.744). Burnout was associated with a positive screen for depression (38.6% vs 12.1%, p=0.011) and lower career satisfaction (77.3% vs 97.0%, p=0.02). EPs with high burnout were significantly more likely to report performing all six acts of suboptimal care.

**Conclusion:**

A majority of EPs demonstrated high burnout. EP burnout was significantly associated with higher frequencies of self-reported suboptimal care. Future efforts to determine if provider burnout is associated with negative changes in actual patient care are necessary.

## INTRODUCTION

Burnout is a triad of emotional exhaustion, depersonalization and reduced sense of personal accomplishment that produces decreased effectiveness at work.^1^ Physician burnout is widespread, with almost half of all physicians reporting high levels of burnout.^2^ Among all specialties, emergency medicine (EM) experiences the highest levels of physician burnout at over 60%.^2^,^3^

High levels of burnout may negatively impact the quality of care physicians provide to patients. Prior work in select medical specialties suggests that burnout is associated with self-reported medical error (e.g., medication errors) and suboptimal care (e.g., failure to adhere to practice standards, lower patient satisfaction).^4^–^14^ Burnout may also contribute to job turnover, absenteeism, low morale, and deterioration of provider health.^3^,^15^–^26^ Although emergency physicians (EPs) report some of the highest levels of burnout, to our knowledge the relationship between EP burnout and patient care has not been studied. Our study evaluated rates of burnout among attending and resident EPs and examined the relationship between their levels of burnout and self-reported patient care practices.

## METHODS

### Study Design

A cross-sectional survey of EPs measured provider levels of burnout and self-reported rates of suboptimal care.

### Study Setting and Population

All attending and post-graduate year (PGY) 2–4 resident EPs, except the study authors, at two university-based PGY 1–4 training programs were eligible for this study conducted in September 2013. PGY-1 residents were excluded from the study because the survey asked respondents to rate their perception of patient care over the past year, and PGY-1 residents at the time of the study had only been in their positions for three months.

### Study Protocol

An anonymous electronic survey was emailed to all eligible subjects. The invitation did not mention burnout, depression, or suboptimal care and subjects were blinded to any specific hypothesis of the study. Subjects consented to the voluntary study by completing the anonymous survey on an online and secure platform (REDCap). Up to two reminder emails were sent to non-responders. The human subjects review boards at both institutions approved the study.

### Measurements

The survey included 39 items taken from previously described instruments on provider burnout, depression, and suboptimal care. Burnout was measured through the Maslach Burnout Inventory (MBI), a 22-item questionnaire that is a standard tool for measuring burnout.^1^,^27^ The MBI evaluates the three dimensions of burnout: depersonalization, emotional exhaustion, and sense of low personal accomplishment. Consistent with prior work, burnout was defined by high scores in the depersonalization or emotional exhaustion subscales of the inventory.^27^ In addition to burnout and depression, we evaluated quality of life (QOL) and career satisfaction. Provider depression was screened using the first two items of the Primary Care Evaluation of Mental Disorders instrument.^28^ A “yes” response to either question was considered a positive screen for depression. We measured QOL by a single-item linear analog scale assessment: “How would you rate your overall quality of life over the past week?”^29^ We assessed career satisfaction by a single-question: “If given the opportunity to revisit your career choice, would you choose to become a physician again?”^30^ Responses of “likely” and “very likely” on a 5-point Likert scale were categorized as positive for career satisfaction.

We measured suboptimal care with a series of six statements adapted from prior work that investigated self-reported patient care among internal medicine resident physicians.^8^ A group of board-certified EPs modified the statements to present EM-focused patient care practices that are common, relevant and important to a practicing EP. The six statements were (1) “I admitted or discharged patients to make the emergency department (ED) more manageable;” (2) “I did not fully discuss treatment options or answer a patient’s questions;” (3) “I ordered more laboratory or radiology tests because I was so busy;” (4) “I did not treat a patient’s pain in a timely manner;” (5) “I did not communicate important information during handoff to an ED colleague or admitting service;” and (6) “I did not discuss a patient’s treatment plan with the patient’s appropriate nursing or ancillary staff.” EPs were asked if they performed these acts of suboptimal care rarely, monthly or weekly over the past year.

To encourage study participation and honest reporting, we collected limited demographic information (Table 1) so that subject responses could not be easily identified. We did not obtain information regarding the subject’s work or training institution.

### Data Analysis

We categorized burnout data as described above, and burnout was dichotomized and defined as meeting the MBI criteria of high emotional exhaustion or high depersonalization.^27^ Burnout, depression, career satisfaction, QOL, and rates of self-reported suboptimal care were compared to career stage (resident versus attending). We then compared burnout to depression, career satisfaction, and self-reported suboptimal care. Comparisons were made using Fischer’s exact test for categorical variables, and Student’s t-test was used for continuous variables. We performed data analysis using STATA version 13 (College Station, TX).

## RESULTS

A total of 91 out of 155 (58.7%) subjects responded to the survey with 77 completed responses included in the analyses (49.7%). Respondents were primarily attending EPs at a university hospital (61.0%), followed by residents (29.9%), and attending EPs at a community hospital (9.1%) (Table 1). EPs reported a burnout rate of 57.1%, with no statistically significant difference between attending and resident physicians. Residents, however, were more likely to report higher scores on the depersonalization subscale than attendings (73.9% vs 38.9%, p=0.011). There were no associations between burnout and gender and year in practice or training. Residents were more likely to screen positive for depression (47.8% vs 18.5%, p=0.012) and report lower QOL scores (6.7 vs 7.4 out of 10, p=0.036) than attendings (Table 2). Attendings and residents reported similar rates of career satisfaction (85.2% vs 87.0%, p=0.744). EP burnout was significantly associated with a positive screen for depression (38.6% vs 12.1%, p=0.011) and lower career satisfaction (77.3% vs 97.0%, p=0.02) (Table 3).

**Figure 1:**
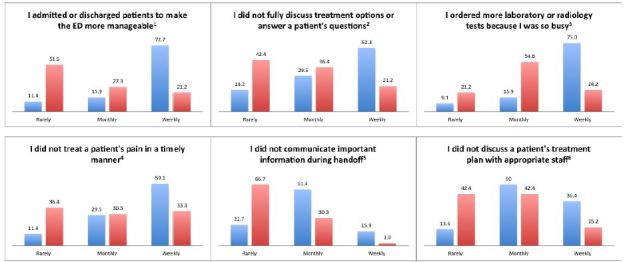
Percentage of emergency physicians and their self-reported frequencies of suboptimal care by burnout. Blue=Burnout; Red=No burnout. ^1^p<0.001; ^2^p=0.012; ^3^p<0.001; ^4^p=0.019; ^5^p<0.001; ^6^p=0.009.

EPs with high levels of burnout were significantly more likely to report performing suboptimal care practices with greater frequency in all six domains (Figure 1): (1) admitting or discharging patients early (p<0.001); (2) not discussing options or answering questions (p=0.012); (3) ordering more tests (p<0.001); (4) not treating patients’ pain (p=0.019); (5) not communicating important handoffs (p<0.001); and (6) not discussing plans with staff (p=0.009). There were no significant associations between rates of suboptimal care and depression, QOL or career satisfaction.

## DISCUSSION

To our knowledge this is the first study to examine the relationship between physician burnout and patient care practices in emergency medicine. Burned-out EPs were more likely to report performing on a more frequent basis all of our queried suboptimal patient care practices. Most prior studies on improving patient safety and quality in emergency medicine have focused on system-level issues rather than individual-level factors.^31^,^32^ Our results suggest that addressing physician factors such as emotional distress and burnout may be important in efforts to improve patient care.

Burnout was common among EPs, with 57% of attending and resident physicians experiencing burnout, a figure that is consistent with studies dating back to 1996.^3^,^24^,^33^ Our study showed no significant difference in levels of burnout between attending and resident EPs. This is similar to a prior study of EPs, which demonstrated that PGY 2–4 residents exhibited burnout rates comparable to those of attending physicians (49–64% vs 60%).^3^ Our findings demonstrate that at least half of EPs suffer from burnout in as early as the second year of residency training. We did not expect burnout rates among attending EPs to be significantly different than those noted in prior studies, since to our knowledge no large organized effort has been made to improve the working conditions of practicing EPs. However, despite efforts to improve resident working conditions, including work hour restrictions, resident burnout has not changed over the last two decades.^33^,^34^

Although our study did not investigate specific causes of EP burnout, previous studies may provide insights into possible explanations for the high rate of EP burnout. Emergency medicine is challenging physically and emotionally.^35^ An unpredictable workload, frequent disruptions to circadian rhythms, and caring for high acuity and high complexity patients in a high stakes environment all potentially contribute to burnout. A national survey of physicians across all specialties found that burnout was highest not just in EM but also in other “front line” disciplines such as general internal medicine and family medicine.^2^ Our results suggest that resident physicians in as early as the PGY-2 level may suffer high levels of burnout similar to those of attending physicians. This may be the result of their socialization in the “hidden curriculum,” a phenomenon in which physicians in training acquire and model the attitudes and habits of other physicians.^36^,^37^ In this sense direct interaction and long hours spent with burned-out EPs may lead to a “contagious” spread of burnout to trainees.

Resident physicians in our study were significantly more likely to report high scores in the depersonalization subscale of the MBI than attending physicians. This is consistent with prior work among residents and attendings across multiple specialties.^38^ Depersonalization is characterized by negative, cynical and dehumanized attitudes and feelings about patients.^39^ We suspect EM residents may experience higher rates of depersonalization due to the fact they on average work more clinical hours than EM attendings at the two sampled academic training sites. In addition resident physicians at these sites are charged with the role of interfacing primarily with admitting services, consultants and ancillary staff to a greater extent than attending physicians. As such resident physicians may experience greater exposure to negative, cynical or dehumanized attitudes about patients.

Our results showed that a positive screen for depression was significantly associated with higher rates of burnout. Burnout is related to depression, although the two are not synonymous.^40^,^41^ Whereas depression affects an individual globally, burnout is specifically related to one’s work. While research on rates of depression among EM residents is limited, our rates of depression among attending physicians are comparable to those in prior studies of EPs.^42^–^44^ Our rates of career satisfaction among EPs were also similar to those reported in previous work,^45^,^46^ as was our study’s demonstrated significant association between burnout and low career satisfaction.^3^,^33^ Interestingly, we did not find significant relationships between suboptimal care and EP rates of depression, QOL or career satisfaction. Although related studies showed associations between various aspects of provider wellness (e.g. burnout, depression, QOL) and physician self-reported medical error,^4^–^6^ only burnout demonstrated a significant relationship with suboptimal care in a similar study of internal medicine trainees.^8^ We theorize that burnout may be a unique and pervasive condition that not only adversely impacts the occurrence of discrete and perhaps more salient medical errors but also the less apparent aspects of quality care (e.g. empathy, professionalism) that physicians provide to patients.

## LIMITATIONS

Our subject population was a convenience sample of EM attending and resident physicians at two academic programs. As such, our results may not be generalizable to EPs in non-academic settings. Approximately 50% of eligible subjects were included in the final analysis, which could allow for response bias. We were unable to compare characteristics of respondents with non-respondents due to the anonymous nature of the survey methodology. Specifically we do not know if non-respondents suffered higher levels of burnout, for example, and therefore did not choose to participate in the study. Still, our rates of burnout are consistent with those reported in prior studies of both academic and non-academic EPs.^2^,^3^,^24^,^33^ Although our questions measuring self-reported suboptimal care were modeled after prior work^8^ and have face validity, their criterion and construct validity as well as reliability have not been examined. In addition we are unable to ascertain if these self-reported frequencies of suboptimal care translate into actual practice. It also remains unclear if burned-out EPs report higher rates of suboptimal care as a result of their higher levels of burnout.^5^,^6^ Despite these limitations, our results are consistent with prior work in other specialties demonstrating that provider wellness, one aspect of which is professional burnout, may significantly impact the quality of care received by patients.^4^–^8^,^12^,^13^,^47^–^50^

## CONCLUSION

A majority of EPs reported high levels of burnout. EP burnout was also significantly associated with higher frequencies of self-reported suboptimal care. Future efforts to determine if provider burnout is associated with negative changes in actual patient care are necessary.

## Figures and Tables

**Table 1:** Demographics of participants in study examining rates of burnout among emergency physicians.

	N (77)	%
Female	29	37.7
Attendings	54	70.1
Years in practice		
<1 yr	8	10.4
1–4 yr	13	16.8
5–10 yr	17	22.1
11–20 yr	8	10.4
21+ yr	8	10.4
Residents	23	29.9
Post-graduate year		
2	7	9.1
3	7	9.1
4	9	11.7
% effort to clinical practice (attendings)		
0–25%	0	0
26–50%	12	22.2
51–75%	19	35.2
76–100%	23	42.6
Primary practice site setting (attendings)		
Academic	47	87.0
Community	7	13.0
Primary practice site annual patient volume		
<25,000	0	0
25,001–50,000	8	10.4
50,001–75,000	24	31.1
75,001–100,000	35	45.5
>100,000	10	13.0

**Table 2:** Rates of provider distress.

	Attending (%)	Resident (%)	Total (%)
Burnout	27 (50.0)	17 (73.9)	44 (57.1)
EE Median (IQR)	20 (12–26)	20 (13–24)	20 (13–26)
Low	21 (38.9)	7 (30.4)	28 (36.3)
Intermediate	21 (38.9)	12 (52.2)	33 (42.9)
High	12 (22.2)	4 (17.4)	16 (20.8)
DP Median (IQR)	10 (7–14)	17 (12–21)	12 (7–19)
Low	13 (24.1)	4 (17.4)	17 (22.1)
Intermediate	20 (37.0)	2 (8.7)	22 (28.5)
High	21 (38.9)*	17 (73.9)*	38 (49.4)
PA Median (IQR)	41 (37–44)	43 (41–44)	42 (38–44)
Low	6 (11.1)	0 (0)	6 (7.8)
Intermediate	13 (24.1)	4 (17.4)	17 (22.1)
High	35 (64.8)	19 (82.6)	54 (70.1)
Depression	10 (18.5)^	11 (47.8)^	21 (27.3)
Career satisfaction	46 (85.2)	20 (87.0)	66 (85.7)
Quality of life (median, IQR)	7.4 (6.4–8.1)#	6.7 (5.8–7.3)#	7.2 (6.1–8.0)

Maslach Burnout Inventory subscales: *EE*, emotional exhaustion; *DP*, depersonalization; *PA,* personal accomplishment

* p=0.011.

^ p=0.012.

# p=0.036.

**Table 3:** Relationship between burnout and depression, career satisfaction.

	Depression	Career satisfaction
Burnout		
Yes (%)	17 (38.6)	34 (77.3)
No (%)	4 (12.1)	32 (97.0)
	p=0.011	p=0.020
